# Identification of Nephrogenic Therapeutic Biomarkers of Wilms Tumor Using Machine Learning

**DOI:** 10.1155/2021/6471169

**Published:** 2021-08-09

**Authors:** Hanxiang Liu, Chaozhi Tang, Yi Yang

**Affiliations:** ^1^Pediatric Urology, Shengjing Hospital of China Medical University, Shenyang 110001, China; ^2^Department of Urology, The First Affiliated Hospital of China Medical University, Shenyang 110001, China

## Abstract

Wilms tumor is the most common renal malignancy in children, with a survival rate of more than 90%; however, treatment outcomes for certain patient subgroups, such as those with bilateral and recurrent diseases, remain significantly below this survival rate. Therefore, it remains essential to identify new biomarkers and develop effective therapeutic strategies. Based on the Therapeutically Applicable Research to Generate Effective Treatments and Gene Expression Omnibus RNA microarray datasets, we have identified eight differentially expressed genes in Wilms tumors as renal-specific in 33 randomly selected adult tumors. The risk model, constructed using survival forest and multivariate Cox regression, can effectively predict the prognosis; the risk score is an independent prognostic factor in Wilms tumor. Gene set enrichment analysis showed that most of the signature genes were involved in regulating human development-related pathways. At the same time, patients in the high-risk group exhibited more sensitive immunological and chemotherapeutic properties than those in the low-risk group. These results provide new insights into personalized and precise Wilms tumor treatment strategies.

## 1. Introduction

Wilms tumor is the most common malignant kidney tumor and the fourth most common cancer in children [1] with an onset age between 3 and 5 years. Approximately, 650 new cases are annually reported in the United States [2]. Histologically, Wilms tumor mimics the various stages of stem cells in the kidney, suggesting the abnormal differentiation of pluripotent mesenchymal stem cells. The classic Wilms tumor comprises three cell types, namely, blastoderm, stroma, and epithelium, which do not coexist in all cases. Meanwhile, Wilms tumors can be divided into two histologic types, namely, “favorable” and “unfavorable.” Approximately, 90% of all cases show a “favorable” histology, including the three abovementioned cell types, and usually have a good prognosis [3]. Conversely, cases with an “unfavorable” histology show a higher degree of dysregulation, which is defined as a polychromatic, polymorphic nucleus that is three times the size of neighboring cells with an abnormal mitotic morphology.

Although the guidelines of the International Society of Pediatric Oncology (SIOP) and the American Pediatric Oncology Group (COG) differ regarding the treatment and risk stratification strategies, their therapies follow relatively simple methods, with very similar outcomes and overall survival rates >85% [4, 5]. Surgery is usually the first line of treatment for Wilms tumor, followed by systemic chemotherapy; however, in some cases, chemotherapy may proceed nephrectomy. Radiotherapy and chemotherapy can effectively improve the survival rate of patients with advanced Wilms tumors; however, they may also increase the risk of secondary malignant tumors years later [6, 7].

Chemotherapeutic agents including actinomycin, adriamycin, and vincristine can increase the risk of secondary malignant tumors, are associated with specific toxicities, may interfere with hearing (carboplatin) and cardiac function (doxorubicin), and may induce peripheral neuropathy (vincristine) [5]. Therefore, identifying new biomarkers for Wilms tumors, reducing the intensity of treatment for children with a low risk of recurrence and developing strategies to reduce their toxicity are urgently needed.

In this study, we have identified unique biomarkers of Wilms tumor using the Therapeutically Applicable Research to Generate Effective Treatments (TARGET) and Gene Expression Omnibus (GEO) RNA-seq datasets and clinical information and verified them using adult kidney tumors. Furthermore, we systematically analyzed the risk characteristics constructed by these biomarkers to predict the prognosis of patients with Wilms tumor and determine their feasibility for immunotherapy and chemotherapy.

## 2. Materials and Methods

### 2.1. Datasets

The RNA-seq transcriptome data, methylation data, and clinical information of the patients were downloaded from the TARGET database (https://ocg.cancer.gov/programs/target), which contains a total of six cases: normal samples, 130 cancer samples, 117 methylated samples, and 125 patients with complete clinical information. The microarray data of the other two datasets, GSE73209 and GSE66405, were downloaded from the GEO database. GSE66405 contains 28 Wilms tumor samples and four normal kidney tissue samples. GSE73209 includes 32 Wilms tumor samples and four fetal kidney tissues for genetic difference analysis. All datasets were processed using the log2 normalization standard. The TCGA pan-cancer RNA sequencing data were downloaded from the UCSC Xena database (https://xenabrowser.net/datapages/), and 18 cancers containing both normal and tumor samples were used for the verification of differentially expressed genes in the Wilms tumors. The comparison of the methylation levels between the normal and tumor samples of kidney renal clear cell carcinoma (KIRC) and kidney renal papillary cell carcinoma (KIRP) was downloaded from the UALCAN (http://ualcan.path.uab.edu) database.

### 2.2. Differentially Expressed Gene Analysis

We used the edgr package at the same time to control the normal and tumor samples of TARGET, GSE66405, and GSE73209 after log2 normalization and used |log2FC| > 1 and *p* < 0.05, as the threshold values to screen for differentially expressed genes. In addition, we analyzed the intersection of the three datasets to obtain 48 differentially expressed genes and used GO (gene ontology) and KEGG (Kyoto Encyclopedia of Genes and Genomes) for functional enrichment analysis.

### 2.3. Random Survival Forest

To identify the differentially expressed genes with significant impacts on the prognosis of Wilms tumors, a univariate Cox proportional hazard regression model was established for each DEG using survival data. We used the coxph function in the Survival *R* package and found that a screening criterion of *p* < 0.05 would not be sufficient to screen the target gene; therefore, we set the threshold to *p* < 0.9. We used the randomSurvivalForest algorithm to rank the importance of prognostic-related genes (ntree < 1,000, which indicates that the number of random forest CHAID decision trees is lower than 1,000). We identified genes with a relative importance >0.3, as the final feature.

### 2.4. Multivariate Regression Was Used to Build a Predictive Model

We performed multiple regression analysis on the eight genes obtained from the random forest algorithm and then obtained their coefficients, *p* value, and *Z* score. The final risk model is as follows:

(1) $$\begin{array}{c} \text{risk score}=0.59190\times \text{UMOD expression}+0.76363\times \text{NAT}8\text{ expression}+\;\\ 0.05435\times \text{CLDN}19\text{ expression}-0.02884\times \text{TMPRSS}2\text{ expression}-0.66439\times \\ \text{DEFB}1\text{ expression}-0.59586\times \text{KCNJ}1\text{ expression}-0.39844\times \text{AQP}2\text{ expression}-\\ \;0.16687\times \text{HMGCS}2\text{ expression. } \end{array}$$

### 2.5. Gene Set Enrichment Analysis (GSEA) of Risk Models

According to the risk score calculated using the above formula, the TARGET patients were divided into high- and low-risk groups, based on the median value. To analyze the significantly enriched pathways between them, we used h.all.v7.4.symbols of GSEA 4.0.1. gmt [Hallmarks], c2.cp.kegg.v7.4.symbols.gmt [Curated], and c5.all.v7.4.symbols.gmt [Gene ontology] as reference datasets, and a *p* < 0.05 was used as the screening threshold. In addition, the gene set related to development was also obtained from the GSEA database (http://www.gsea-msigdb.org/gsea/index.jsp).

### 2.6. Chemical Prediction and Immune Prediction

We used the *R* package “pRRophetic” to predict the chemotherapy response of each sample through a 10-fold crossover based on the GDSC training set and verified the accuracy of the prediction. The half-maximal inhibitory concentration (IC50) of the sample was estimated using ridge regression. Second, based on the data on the immune response of melanoma patients who have recently received checkpoint blockade treatment against cytotoxic T lymphocyte antigen 4 (CTLA-4) and programmed death receptor 1 (PD-1) [8], we used the subgraph method to predict the responsiveness of the high- and low-risk groups to immunotherapy [9].

### 2.7. Prediction of Drug Susceptibility of Signature Genes

We downloaded the Compound activity: DTP NCI-60 and the RNA: RNA-seq data from the CellMinerCDB (Version: 2020.4) database (https://discover.nci.nih.gov/cellminer/home.do). Only clinical trials and FDA-approved drugs were retained, the correlation between the signature genes and drug sensitivity was checked, and a Spearman correlation coefficient >0.3 and a *p* < 0.05 were used as the screening thresholds.

### 2.8. Statistical Analysis

All statistical tests were performed using *R* version 4.0.3. The Wilcoxon test was used for the comparison between two groups of data. A nonparametric test was used for Spearman correlation analysis within the study, and log-rank test was used for the survival analysis of the Kaplan–Meier curve. Bonferroni correction was used to adjust the immunotherapy response in the high- and low-risk groups. All tests were two-sided, and statistical significance was set at a *p* value < 0.05.

## 3. Results

### 3.1. Identification of Differential Genes in Wilms Tumors

**Figure 1:**
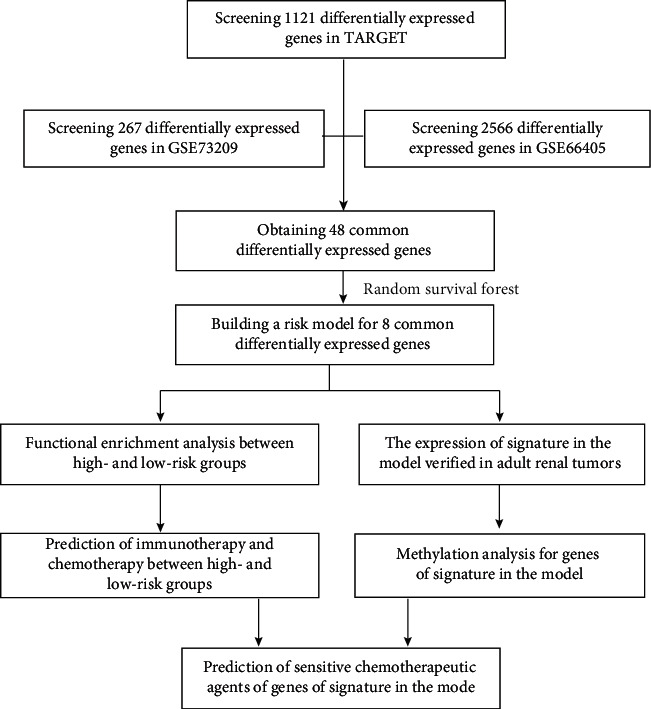
Flow chart of this study.

**Figure 2:**
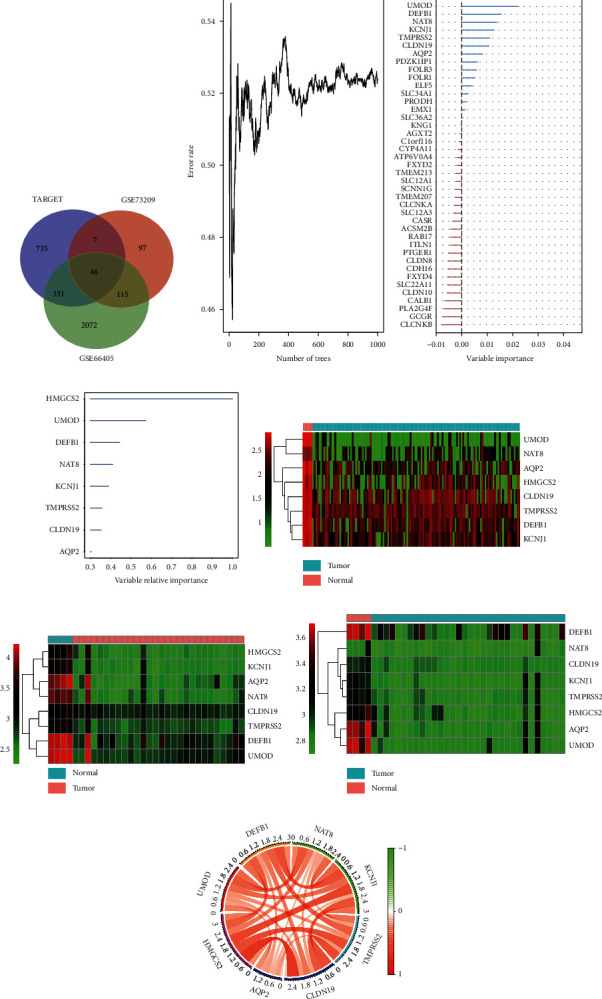
Identification of differential genes in Wilms tumor. (a) Venn diagram, (b) error rate, and variation importance of the 48 differentially expressed genes using dimensionality reduction analysis of random survival forest. (c) Target genes with a relative importance > 0.3 of the random survival forest. Heat map of the eight differentially expressed genes finally identified in normal and tumor tissues in (d) TARGET, (e) GSE66405, and (f) GSE73209.

First, in order to screen the important differentially expressed genes, we used the edgr package to compare the normal and tumor samples using TARGET and GSE73209 datasets after log2 normalization, and |log2FC| > 1 and *p* < 0.05 were used as the threshold values to obtain 1,121 differentially expressed genes. By intersecting these with 2,567 genes derived from GSE66405, 48 differentially expressed genes were obtained (Figures 1 and 2(a)).

### 3.2. Random Survival Forest

To identify genes with significant effects on prognosis of the 48 differentially expressed genes, we used the randomForestSRC *R* software package for functional selection. We identified genes with a relative importance >0.3 as the final feature.  Figure 2(b) depicts the relationship between the error rate and the number of classification trees, and Figure 2(c) shows the final order of relative importance of the first eight genes. Figures 2(d)–2(f) show the expression of these eight genes, *HMGCS2*, *UMOD*, *DEFB1*, *NAT8*, *KCNJ1*, *TMPRSS2*, *CLDN19*, and *AQP2*, in the normal and cancer tissues of the TARGET, GSE66405, and GSE73209 datasets. Compared with the normal tissues, these eight genes were consistently downregulated in cancer tissues and were expressed as a significant positive correlation (Figure 2(g)).

### 3.3. Construction of a Prognostic Risk Model Based on the Differentially Expressed Genes

Following the identification of eight genes using random survival forest, we conducted multiple regression analysis to construct a risk model for the prognostic evaluation of Wilms tumor patients.  Table 1 shows the coefficients, *p* value, *Z* score, raw importance, and relative importance of these eight genes.

**Figure 3:**
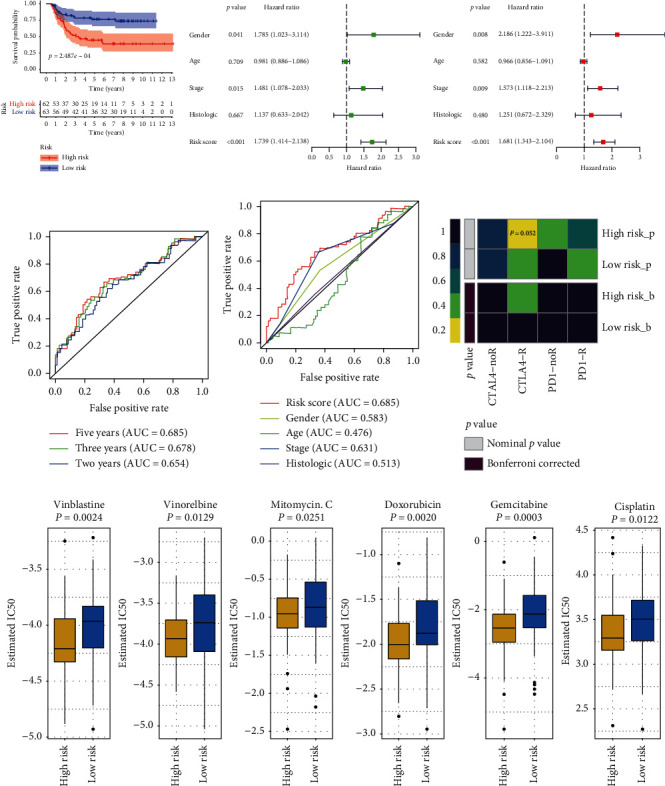
Clinical characteristics and immunochemical predictive analysis of the risk model. (a) Analysis of the Kaplan–Meier survival curve based on the risk model. (b) Univariate and (c) multivariate analysis of the risk score and clinical characteristics. (d) The high- and low-risk group responsiveness to treatment is based on the data of the immune response of melanoma patients who had recently received checkpoint blockade treatment against cytotoxic T lymphocyte antigen 4 (CTLA-4) and programmed death receptor 1 (PD-1). (e) The chemical prediction response of the high- and low-risk group patients is based on the trained model used on the GDSC cell line dataset.

According to the median risk score, patients with Wilms tumors were divided into high- and low-risk groups. The survival rate of patients with low-risk scores was much higher than that of patients in the high-risk group (Figure 3(a)). At the same time, to evaluate the relationship between the validity of the risk model and the clinical characteristics, we analyzed the predictive relationship between the age, sex, stage, histological type, and risk score from the perspective of single factors and multiple factors. Differences in sex, stage, and risk score were statistically significant as indicated using the univariate and multivariate regression analyses (*p* < 0.05), suggesting that they are opposite prognostic factors for patients with Wilms tumor. In addition, we used the *R* software package timeROC to perform receiver operating characteristic (ROC) analysis of the prognostic classification of the risk score and analyzed the classification efficiency of the 2-year, 3-year, and 5-year prognostic predictions. The ROC curve showed that the 2-year, 3-year, and 5-year AUCs were 0.654, 0.678, and 0.685, respectively. Similarly, the 5-year risk score AUC of patients with Wilms tumors was better than that of the sex, age, stage, and histology (Figure 3(e)).

### 3.4. Chemical Prediction and Immune Prediction

To further explore the possibility and applicability of the risk model constructed in a clinical setting, based on the recent acceptance of cytotoxic T lymphocyte antigen 4 (CTLA-4) and programmed death receptor 1 (PD-1) checkpoint blockade of the immune response data of melanoma patients treated, the subgraph method was used to predict the responsiveness of the high- and low-risk groups to immunotherapy. Patients in the high-risk group were more likely to be treated with CTLA-4. Second, considering that chemotherapy is the most common treatment for patients with Wilms tumor after surgery (Figure 3(f)), we trained the prediction model on the GDSC cell line dataset through ridge regression and evaluated it using a 10-fold cross-validation satisfactory forecast accuracy. We found that the high- and low-risk groups showed highly consistent responsiveness to some commonly used chemotherapeutic agents against Wilms tumors, that is, in the prediction of the sensitivity to vinblastine, vinorelbine, mitomycin C, doxorubicin, gemcitabine, and cisplatin. Furthermore, the high-risk group patients had a significantly higher response sensitivity than the low-risk group patients (Figure 3(g); *p* < 0.05).

### 3.5. Gene Set Enrichment Analysis of the High- and Low-Risk Groups

**Figure 4:**
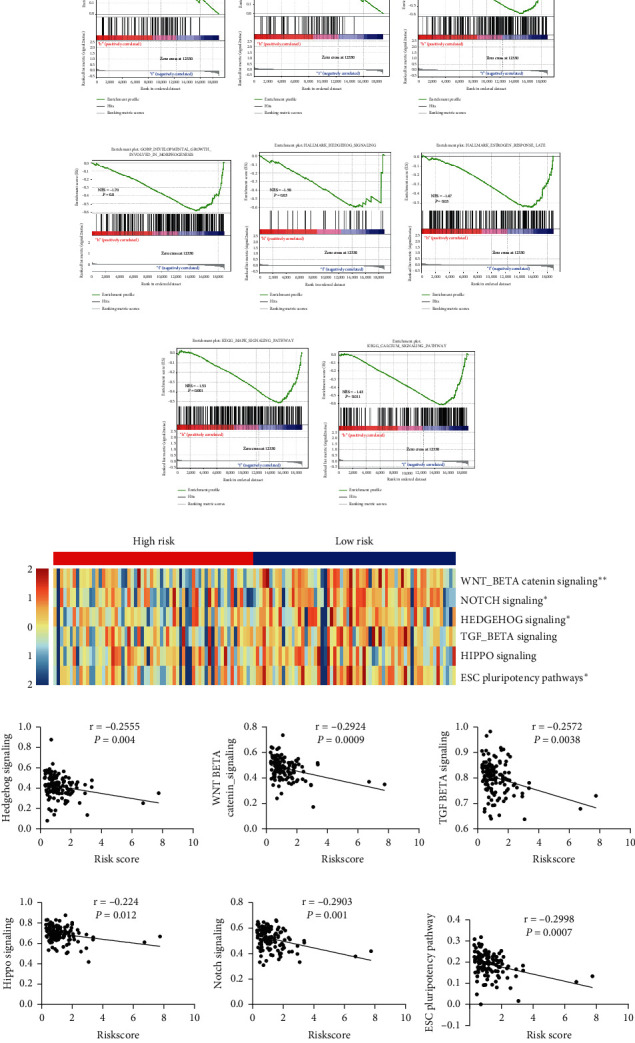
Gene set enrichment analysis. (a–h) Gene set enrichment analysis of the high- and low-risk groups. (i) Heat map of the ssGSEA scores in six developmental pathways of the high- and low-risk groups. (j–o) Correlation analysis of the risk score and ssGSEA score of each developmental pathway. NES: normalized enrichment score. ^*∗*^*p* < 0.05 and ^*∗∗*^*p* < 0.01.

Next, explain these differences in drug sensitivity between the high- and low-risk groups, we performed Gene Set Enrichment Analysis (GSEA) on the high- and low-risk groups to characterize the differences in the functional pathways between the two groups. Compared with the low-risk group, the high-risk group showed significant activation of the ERAD pathway and ubiquitin-dependent ERAD pathway, while in developmental cell growth, morphogenesis, hedgehog signaling, late estrogen response, MAPK signaling pathway, and calcium signaling pathway were significantly inhibited (Figure 4). The high- and low-risk groups manifested significant abnormalities in the development-related pathways. We collected the six major human developmental signaling pathways in the GSEA dataset and quantified their scores in each Wilms tumor sample using the ssGSEA algorithm. The results showed that the high-risk group was associated with Wnt/*β*-catenin signaling, Notch signaling, and hedgehog signaling, compared to the low-risk group. The scores of signaling, TGF-*β* signaling, Hippo signaling, and ESC pluripotency pathway were low. Further, Spearman correlation analysis showed that the risk score was significantly negatively correlated with the ssGSEA scores of these six developmental signaling pathways (Figure 4).

### 3.6. Expression of Signature Genes in Pan-Cancer

**Figure 5:**
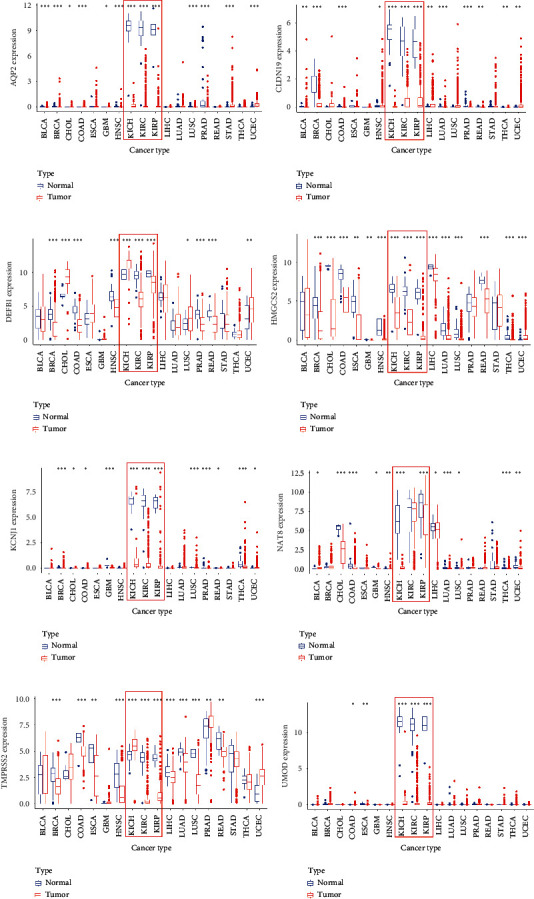
Verification of the expression of eight differentially expressed genes in pan-cancer. (a–h) *AQP2*, *CLDN19*, *DEFB1*, *HMGCS2*, *KCNJ1*, *NAT8*, *TMPRSS2*, and *UMOD* expression in pan-cancer; ^*∗*^*p* < 0.05, ^*∗∗*^*p* < 0.01, and ^*∗∗∗*^*p* < 0.001.

We downloaded 33 types of TCGA cancers from UCSC Xena to examine the expression patterns of the signature genes of adult tumors. The signature genes were significantly downregulated in most tumors. Apart from *DEFB1* and *TMPRSS2*, which were upregulated in KICH, the other genes were significantly downregulated in KICH, KIRC, and KIRP. At the same time, the expression of AQP2, CLDN19, DEFB1, KCNJ1, NAT8, and UMOD in the normal tissues of KICH, KIRC, and KIRP was much higher than that in the normal tissues of other tumors. Therefore, these may be unique signs of renal origin (Figure 5).

### 3.7. Methylation of Signature Genes Was Explored in Adult Renal Cancer

**Figure 6:**
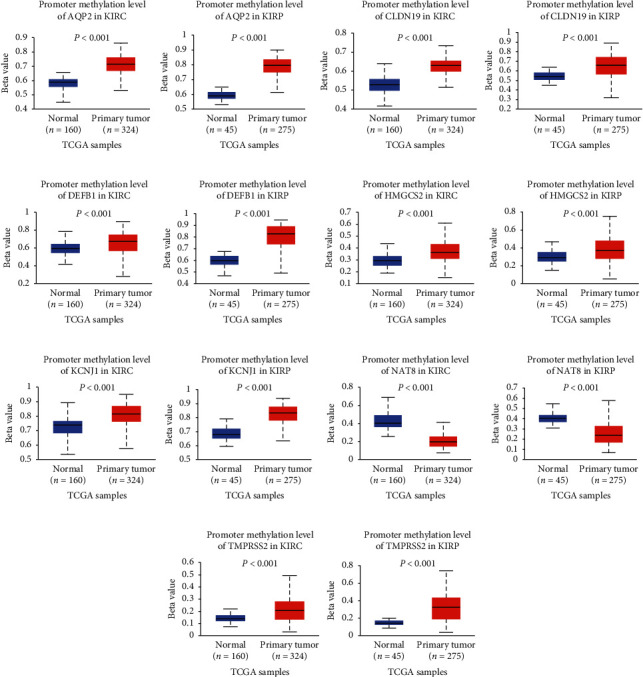
The methylation of the signature genes in adult renal cancer. (a–g) Methylation levels of AQP2, CLDN19, DEFB1, HMGCS2, KCNJ1, NAT8, and TMPRSS2 in normal and tumor tissues of KIRC and KIRP. KIRC: kidney renal clear cell carcinoma; KIRP: kidney renal papillary cell carcinoma.

**Figure 7:**
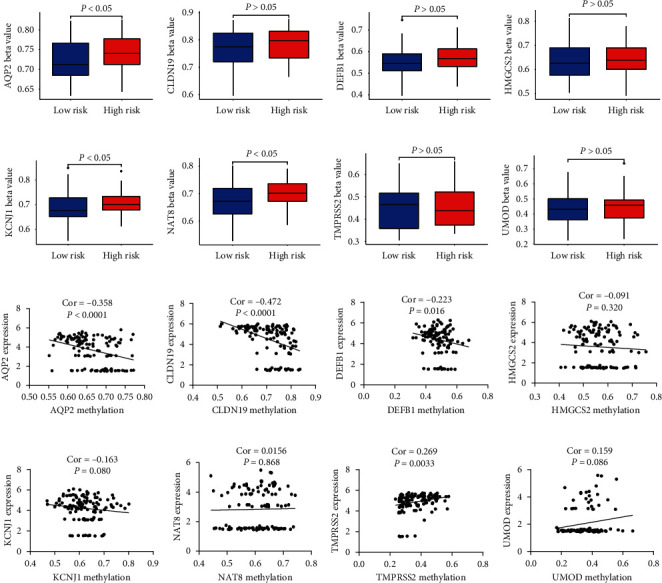
Methylation comparison between the high- and low-risk groups and correlation analysis of the mRNA expression and methylation level. (a–h) Comparison between the high- and low-risk groups regarding the methylation of AQP2, CLDN19, DEFB1, HMGCS2, KCNJ1, NAT8, TMPRSS2, and UMOD. (i–p) Correlation analysis of the mRNA expression and methylation level of AQP2, CLDN19, DEFB1, HMGCS2, KCNJ1, NAT8, TMPRSS2, and UMOD; Spearman correlation.

To further clarify the reasons for the general low expression of the above-mentioned signature genes in adult renal tumors, we used the genes of the query signature, *AQP2*, *CLDN19*, *DEFB1*, *KCNJ1*, *NAT8*, *HMGCS2*, and *TMPRSS2* (the average beta value for “UMOD” is not available for the majority of the samples in KICH, KIRC, and KIRP; KICH has no normal methylation data). The methylation modification results of KIRC and KIRP show that the methylation level of the promoter region of *AQP2*, *CLDN19*, *DEFB1*, *KCNJ1*, *HMGCS2*, and *TMPRSS2* is elevated compared to the normal samples, which indicates their hypermethylation. The methylation level in the promoter region of *NAT8* was significantly reduced, which indicates hypomethylation (Figure 6). At the same time, we compared the methylation modification levels of the high- and low-risk groups in the TARGET dataset and found that in addition to *TMPRSS2*, for the low-risk group, the high-risk group had a higher level of methylation modifications. The mRNA expression levels of AQP2, CLDN19, and DEFB1 were significantly negatively correlated with the methylation modifications, while the mRNA expression levels of TMPRSS2 and UMOD were significantly positively correlated with the methylation modifications, while the relationship between the mRNA expression level of other genes and the methylation modifications remains nebulous (Figure 7).

### 3.8. Prediction of the Sensitivity of Signature Genes in CellMiner Database

**Figure 8:**
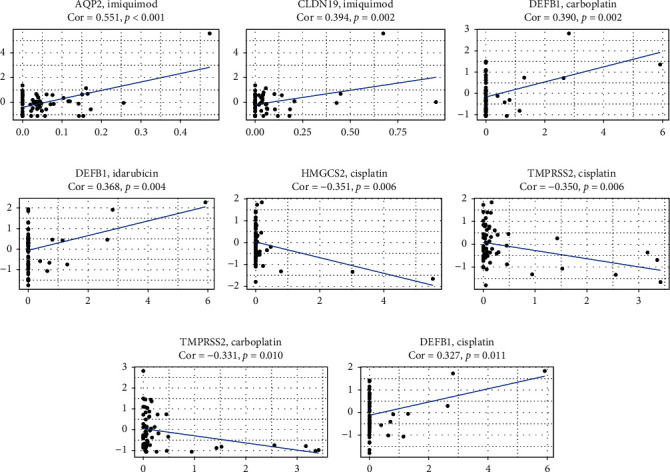
Prediction of the sensitivity induced by the signature genes using CellMiner database. (a–h) The scatter plot indicates the correlation between the target gene expression and drug sensitivity (the Z score of the CellMiner interface) for the Spearman correlation test using NCI-60 cell line data.

To further explore the possibility of clinical application of the signature genes and promote a more precise treatment, we used the CellMiner database to evaluate the impact of the target genes on drug sensitivity. Drug sensitivity was measured using the *Z* score. The higher the score, the more sensitive the cells were to the drug treatment (Figure 8). Our results showed that the expression of AQP2 and CLDN19 was increased, which is associated with the sensitivity to imiquimod. The increased expression of DEFB1 is associated with the sensitivity to carboplatin and idarubicin, while the increased expression of TMPRSS2 and HNGCS2 is associated with resistance to cisplatin.

## 4. Discussion

The etiology of Wilms tumor remains unclear; however, it may be influenced by genetic changes. For example, some genetic biomarkers associated with Wilms tumors have been identified in approximately 1/3 of all Wilms tumors include *WT1*, *CTNNB1*, and *WTX* mutations or the loss of heterozygosity of the 1p, 1q, 11p15, and 16q chromosomes [10] affecting the normal embryonic development of the genitourinary tract. Therefore, Wilms tumor can also be classified as a developmental disease. In this study, we used machine learning methods to screen the differentially expressed genes from multiple datasets and identified eight genes that affect the survival of patients with Wilms tumor. Further, the risk model constructed can also classify patients with Wilms tumor into groups of significantly different risk, to effectively predict prognosis, demonstrating that the risk score is an independent prognostic factor for Wilms tumor. Further verification of GSEA and pan-cancer signature gene expression demonstrates that the signature genes are specifically of kidney origin and are associated with pathways that regulate human development. In particular, developmental cell growth is involved in morphogenesis, hedgehog signaling, Wnt/*β*-catenin signaling, and Notch signaling. Our results showed that as the risk score increased, the corresponding ssGSEA score of development-related pathways decreased; that is, the risk score is significantly negatively correlated with the development-related pathways. It has been reported that an excessive activation of development-related pathways, such as Wnt/*β*-catenin signaling [11–13] and hedgehog signaling [14], can lead to the progression or metastasis of Wilms tumors. Our results also demonstrated that compared with normal tissues, in patients with Wilms tumor, whether in high- or low-risk groups, Wnt/*β*-catenin, Notch, hedgehog, and hippo signaling pathways have different degrees of activation. However, the low-risk group patients had a higher degree of activation of the abovementioned pathways than the high-risk group patients. Therefore, it is difficult to associate the activation of these pathways with an absolute prognosis equivalent of Wilms tumor. We believe that in the high-risk group, the regulation of the activated ERAD pathway, the ubiquitin-dependent ERAD pathway, may be one of the main underlying reasons for the occurrence of Wilms tumors.

ERAD consists of a series of spatiotemporal coupled activities that mediate the recognition of proteins, cytoplasmic delocalization (also called reversal), and the ER-dependent ubiquitin-dependent proteasomal degradation of selected proteins, at physiological levels. In this state, ERAD maintains the quality of the secreted proteome by degrading proteins with mutations, transcription and translation errors, or inability to assemble into natural oligomers. However, an overly strict ERAD system can have catastrophic consequences. The ΔF508 mutant form of the cystic fibrosis transmembrane conductance regulator (CFTR), the most common cause of cystic fibrosis, is retained, despite its local function being effectively degraded by ERAD [15, 16]. The inability of proteins to mature and transport to the plasma membrane can lead to severe chloride transport defects, which manifest as the thickening of mucus in the lungs, pancreas, and other organs. Pharmacological treatment can selectively damage ERAD and promote the maturation of functional mutant proteins, which can be effective against cystic fibrosis and other diseases associated with protein maturity changes [17].

DNA methylation at CpG dinucleotides is the most well-characterized epigenetic marker, which has been largely reprogrammed between different generations of mammals [18]. Although only hundreds of imprinted genes have been described to date, it has been demonstrated that imprinting plays a key role in several biological processes, including development, growth, cell cycle [19], heredity, and circulation. Epigenetic analysis of cell-free DNA (cfDNA) isolated from the blood of patients with Wilms tumor can be used to predict tumor prognosis and monitor responses to treatment and can help diagnose different cancers [20–22]. Pritchard-Jones et al. have analyzed the methylation of cfDNA extracted from the blood of children with or without Wilms tumor and found that the genomic region (DMR-2) close to the *PRRT1* gene was relatively highly methylated before treatment in children with Wilms tumor. In addition, the level of epitope mutations in this genomic region is significantly increased following the preoperative chemotherapy phase and maintained immediately after the operation [23]. Similarly, of the eight signature genes in our study, seven had abnormally high methylation levels in the priming region in adult tumors KIRC and KIRP, while the methylation level of NAT8 in the priming region was significantly reduced. However, this methylation modification is likely to be the reason for their significant downregulation in tumors. Although the TARGET dataset does not contain the methylation data of normal samples, we compared the mRNA and methylation data of the eight signature genes in the high- and low-risk groups. The methylation modifications and mRNA expression were negatively correlated, while at the same time, the correlation analysis between the mRNA expression and the methylation levels also verified that the methylation modifications of the signature genes negatively regulate the mRNA expression.

In addition, in terms of molecular targeted therapy prediction, our results show that the increased expression of AQP2 and CLDN19 is associated with the sensitivity to imiquimod, an imidazoline derivative commonly used to treat genital warts. It was the first small-molecule immuno-oncology drug approved by the FDA for the treatment of basal cell carcinoma [24, 25]. Imiquimod targets toll-like receptor 7 (TLR7), which is a pattern recognition receptor (PRR) that binds to conserved PAMPs, such as double-stranded RNA, lipopolysaccharide, or unmethylated CpG-DNA. Most TLRs are expressed on the cell surface, but TLR3, 7, 8, and 9 are mainly located in endosomes. The small TLR8 agonist motolimod (VTX-2337) exhibits antitumor activity in recurrent or metastatic head and neck squamous cell carcinoma (HNSCC) by stimulating natural killer cells and enhancing antibody-dependent cell-mediated toxicity. Motolimod combined with cetuximab (anti-EGFR antibody), or conventional chemotherapy, can decrease the number of Tregs in the tumor microenvironment and increase in the number of circulating EGFR-specific CD8+ T cells. Compared with cetuximab or chemotherapy, the progression-free survival (PFS) and overall survival rates were increased [26, 27]. Imiquimod, motolimod, and resiquimod (targeting TLR7 and TLR8) are currently undergoing a series of clinical trials (NCT03276832, NCT0397626, NCT02126579, and NCT01204684) for the treatment of solid tumors, usually as adjuvants for vaccination. Therefore, the development of vaccines for AQP2 can further serve the clinical application of Wilms tumor treatments.

However, despite the risk model we constructed, which consists of eight genes, the prognosis and clinical features, immunology and chemotherapy, the correlation between mRNA expression and methylation modifications, the regulation of the developmental pathways, and the development of molecularly targeted drugs have achieved good predictive performance [28, 29]. Since the data on Wilms tumors with complete follow-up information is limited, the prognosis and clinical characteristics of patients with Wilms tumor are also limited in the TARGET database; therefore, studies with larger samples are urgently needed. However, our results suggest these eight gene markers as potential prognostic biomarkers thereby providing new insights into novel therapeutic strategies for Wilms tumors.

## 5. Conclusions

Our results show that the Wilms tumor risk model constructed based on the eight differentially expressed genes with a kidney origin, which were identified in multiple datasets, can not only effectively predict the prognosis of Wilms tumor patients but also expand the immune response to Wilms tumors. Targeting these eight genes as a novel treatment strategy against Wilms tumors warrants further investigations, which may lead to the development of new vaccine for Wilms tumor.

## Figures and Tables

**Table 1:** The coefficients, *p* value, *Z* score, raw importance, and relative importance of these eight genes.

Gene	Coef	z	*p*	Raw importance	Relative importance
UMOD	0.5919	1.612	0.1071	0.02239931	0.57317073
TMPRSS2	−0.02884	−0.048	0.9614	0.01098428	0.35772358
NAT8	0.76363	2.174	0.0297	0.01378419	0.41056911
KCNJ1	−0.59586	−1.271	0.2036	0.01270730	0.39024390
HMGCS2	−0.16687	−0.467	0.6403	0.045014	1
DEFB1	−0.66439	−1.755	0.0793	0.01550722	0.44308943
CLDN19	0.05435	0.158	0.8741	0.01076889	0.35365854
AQP2	−0.39844	−1.396	0.1628	0.00818436	0.30487805

Coef: coefficient; *p*: *p* value; z: *Z* score.

## Data Availability

The datasets generated for this study can be found in the TARGET (https://ocg.cancer.gov/programs/target), GEO (https://www.ncbi.nlm.nih.gov/geo/), and UCSC Xena (https://xenabrowser.net/datapages/) databases.
